# Supplementary material for: The productivity-biodiversity relationship varies across diversity dimensions
Source: Nat Commun. 2019 Dec 12;10:5691. doi: 10.1038/s41467-019-13678-1 (PMC6908676; doi:10.1038/s41467-019-13678-1)
Supplement: Supplementary file 1 — Supplementary Information [file 41467_2019_13678_MOESM1_ESM.pdf]

Supplementary Information

to the paper "The productivity-biodiversity relationship varies across diversity dimensions" by Brun et al.

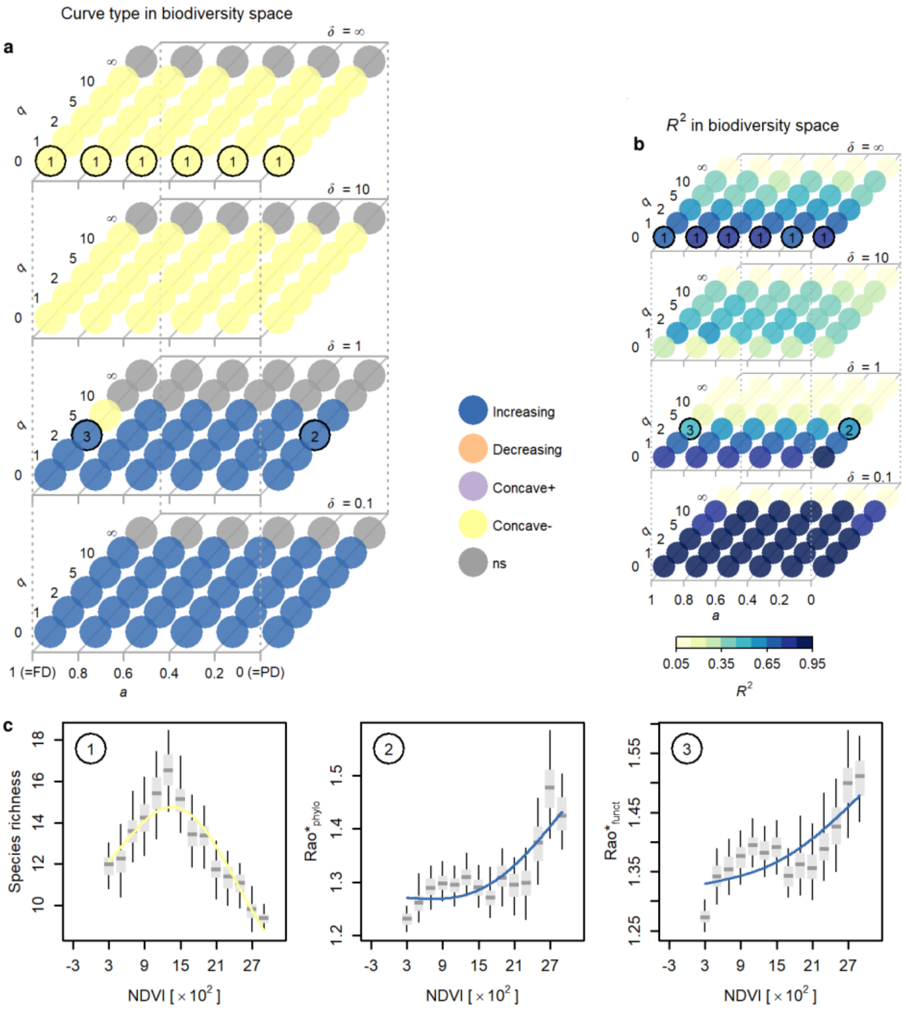

Supplementary Figure 1: Biodiversity responses to NDVI across biodiversity space considering additional traits. In panel (a) shapes of the relationships are summarized across biodiversity space. X-axis corresponds to increasing emphasis on trait relative to phylogenetic information; y-axis represents increasing emphasis on dominant relative to rare species; z-axis represents increasing emphasis on high versus low species similarities. Numbers indicate locations of focal biodiversity metrics within the biodiversity space. Panel (b) shows R^2 of the fits across biodiversity space. Detailed relationships of focal biodiversity metrics are illustrated in panel (c); Central lines in boxplots illustrate medians, boxes illustrate interquartile ranges, and whiskers show 95% confidence intervals. Overlaid are univariate GAM-fits with colors representing type of the curve (see legend in panel (a)). Rao* represents 1/(1-Rao), with Rao being Rao's quadratic entropy[1]. Relationships were estimated based on a subset of *c.* 3300 community plots that were dominated by *c.* 300 species for which trait diversity was estimated considering on the main trait set of height, SLA, and seed mass, as well as LDMC and LNC.

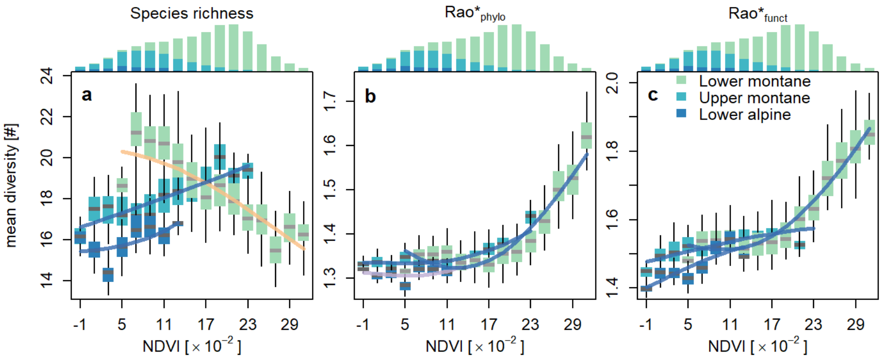

Supplementary Figure 2: Relationships between productivity and focal biodiversity metrics for different climate zones. Climate zones include the lower montane zone (turquoise), the upper montane zone (blue) and the lower alpine zone (dark blue). Central lines in boxplots illustrate medians, boxes illustrate interquartile ranges, and whiskers are 95% confidence intervals. Overlaid curves are corresponding GAM-fits, colored according to curve type classification: blue is increasing; orange is decreasing; and purple is concave. Standard errors are hardly visible in this representation and thus not depicted. Histograms illustrate the frequency distribution of the climate zones along the productivity gradient. Rao* represents 1/(1-Rao), with Rao being Rao's quadratic entropy[1].

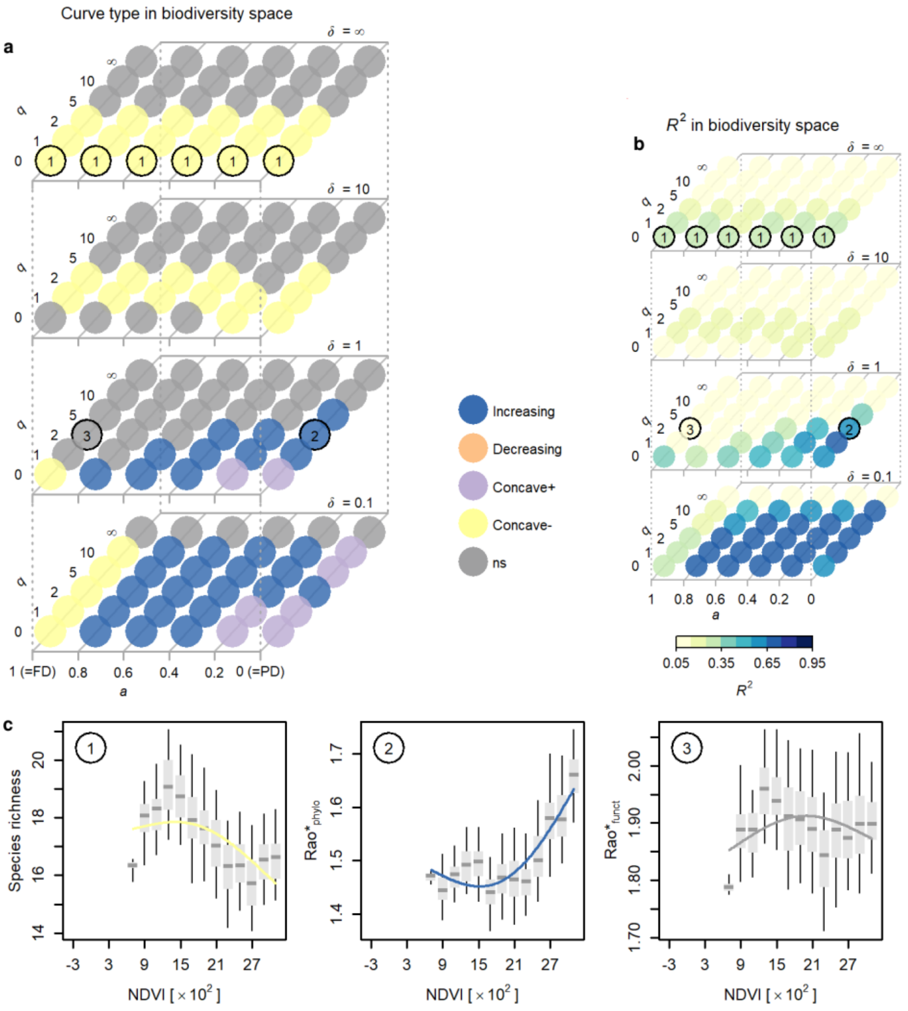

Supplementary Figure 3: Forest biodiversity responses to NDVI across biodiversity space. In panel (a) shapes of the relationships are summarized across biodiversity space. X-axis corresponds to increasing emphasis on trait relative to phylogenetic information; y-axis represents increasing emphasis on dominant relative to rare species; z-axis represents increasing emphasis on high versus low species similarities. Numbers indicate locations of focal biodiversity metrics within the biodiversity space. Panel (b) shows R^2 of the fits across biodiversity space. Detailed relationships for focal biodiversity metrics are illustrated in panel (c). Central lines in boxplots illustrate medians, boxes illustrate interquartile ranges, and whiskers show 95% confidence intervals. Overlaid are univariate GAM-fits with colors representing type of the curve (see legend in panel (a)). Rao* represents 1/(1-Rao), with Rao being Rao's quadratic entropy[1].

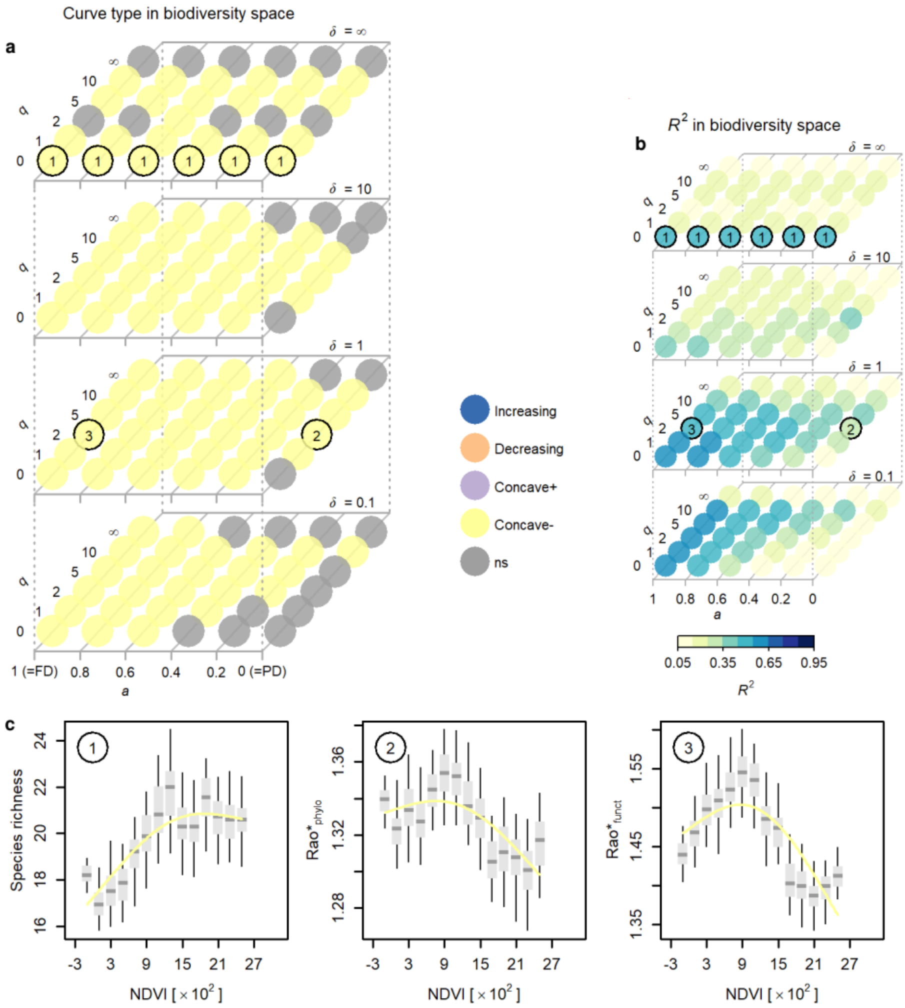

Supplementary Figure 4: Grassland biodiversity responses to NDVI across biodiversity space. In panel (a) shapes of the relationships are summarized across biodiversity space. X-axis corresponds to increasing emphasis on trait relative to phylogenetic information; y-axis represents increasing emphasis on dominant relative to rare species; z-axis represents increasing emphasis on high versus low species similarities. Numbers indicate locations of focal biodiversity metrics within the biodiversity space. Panel (b) shows R^2 of the fits across biodiversity space. Detailed relationships for focal biodiversity metrics are illustrated in panel (c). Central lines in boxplots illustrate medians, boxes illustrate interquartile ranges, and whiskers show 95% confidence intervals. Overlaid are univariate GAM-fits with colors representing type of the curve (see legend in panel (a)). Rao* represents 1/(1-Rao), with Rao being Rao's quadratic entropy[1].

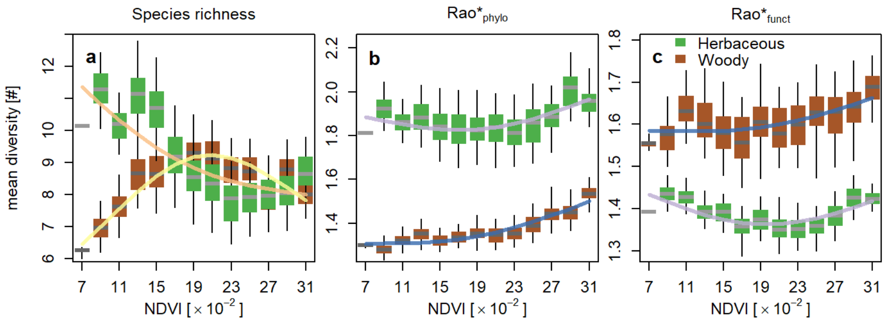

Supplementary Figure 5: Relationships between productivity and focal biodiversity metrics for the woody and the herbaceous parts of forest vegetation. Relationships for woody species are shown in brown; relationships for herbaceous species are shown in green. Central lines in boxplots illustrate medians, boxes illustrate interquartile ranges, and whiskers are 95% confidence intervals. Overlaid curves are corresponding GAM-fits, colored according to curve type classification: blue is increasing; orange is decreasing; yellow is concave- and purple is concave+. Standard errors are hardly visible in this representation and thus not depicted. Histograms illustrate the frequency distribution of the climate zones along the productivity gradient. Rao* represents 1/(1-Rao), with Rao being Rao's quadratic entropy[1].

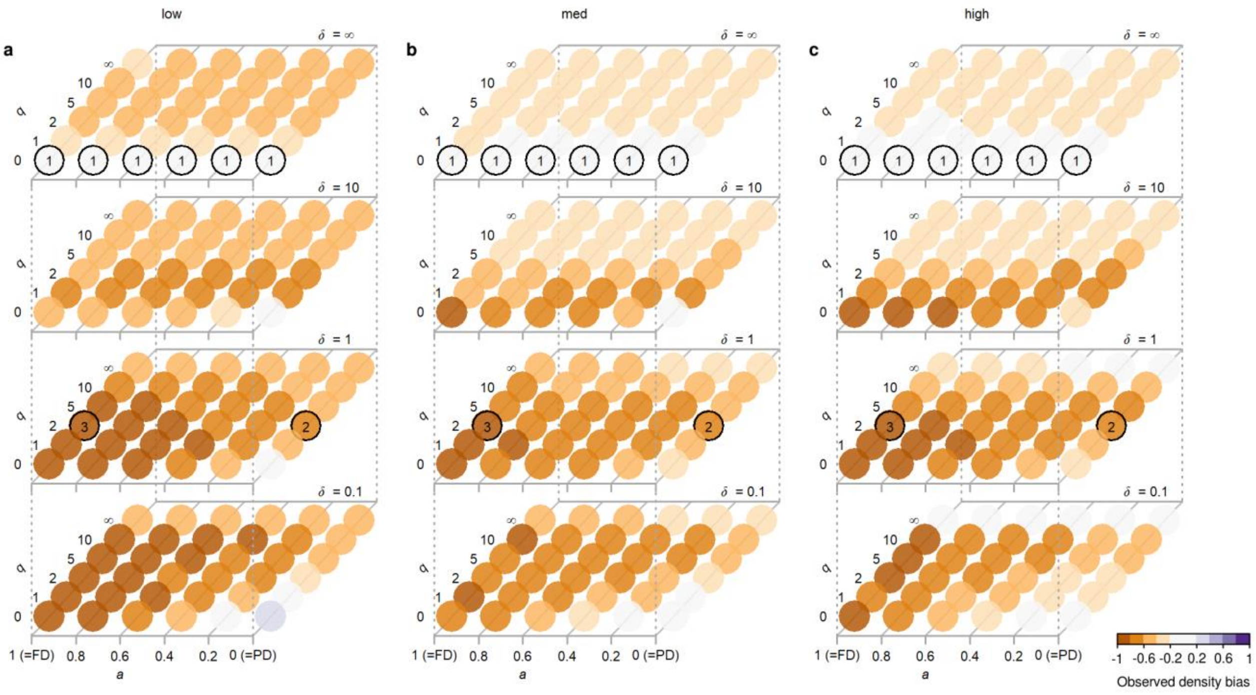

Supplementary Figure 6: Biodiversity in high-productivity grasslands compared to null expectations for different land use intensities across biodiversity space. Differences are shown as mass fraction of high-productivity biodiversity estimates that are higher (positive values) or lower (negative values) than null expectations. Panels represent low (a), medium (b), and high (c) land use intensity. X-axis corresponds to increasing emphasis on trait relative to phylogenetic information; y-axis represents increasing emphasis on dominant relative to rare species; z-axis represents increasing emphasis on high versus low species similarities

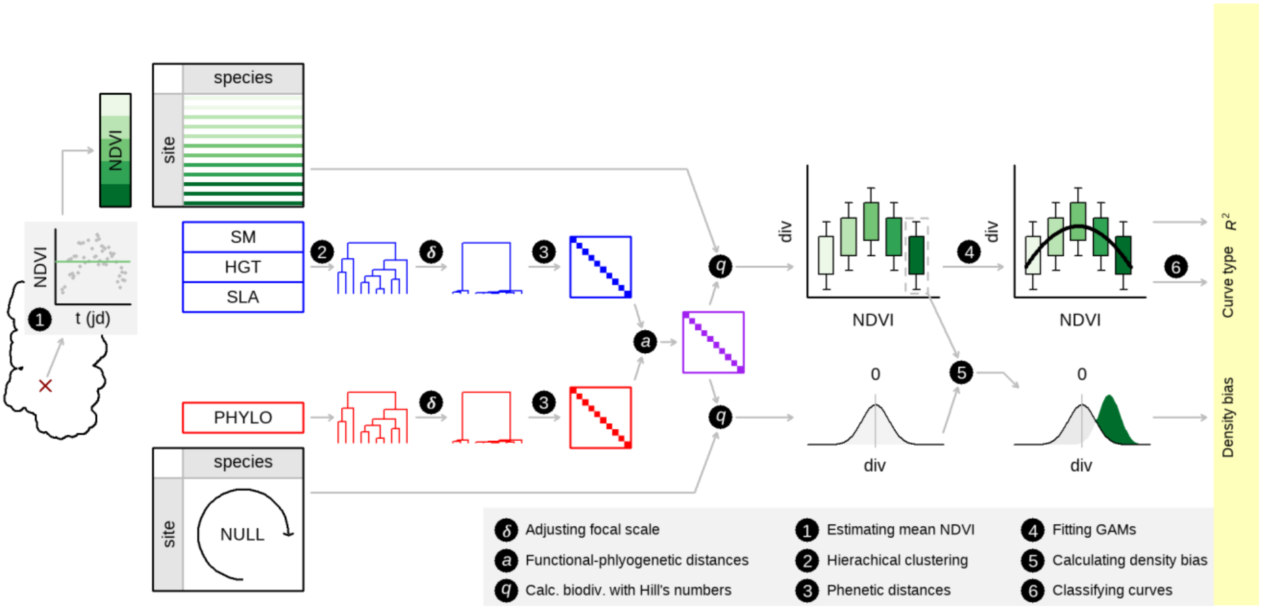

Supplementary Figure 7: Schematic illustration of the analyses conducted in this study. Arrows indicate flow of information; black circles indicate data transformations. Blue represents derivatives from trait information; derivatives from phylogenetic information are shown in red. Green shades indicate increasing levels of NDVI. NULL indicates random assemblages; jd stands for Julian day. Further information is provided in the Methods.

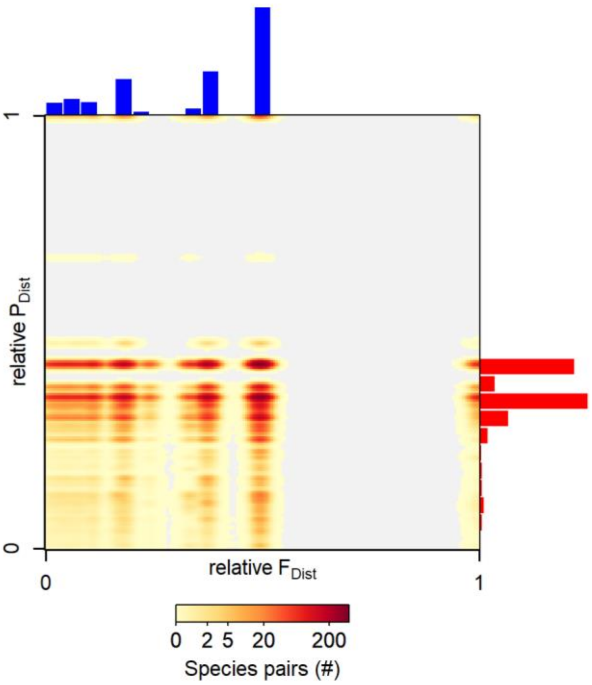

Supplementary Figure 8: Relationship between pairwise distances in functional and phylogenetic trees. The 1’456’071 pairwise combinations functional and phylogenetic distances for the 1210 species are summarized in a density surface. Zero values are depicted in light grey (hardly visible on color scale). Blue histogram on the top depicts the frequency distribution of functional distances; red histogram on the right depicts frequency distribution of phylogenetic distances.

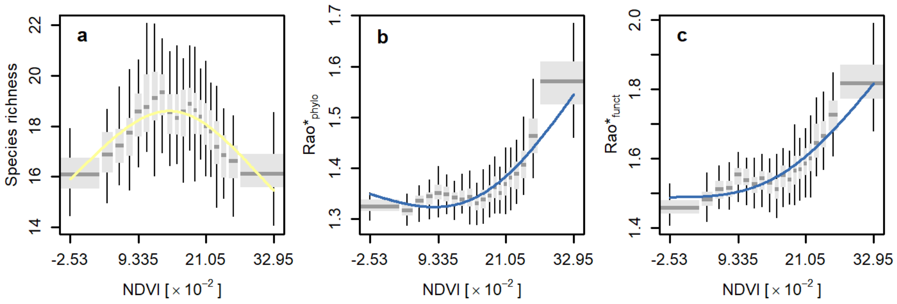

Supplementary Figure 9: NDVI-biodiversity relationships for focal metrics using bin widths defined by 5%-percentiles. Central lines in boxplots illustrate medians, boxes illustrate interquartile ranges, and whiskers show 95% confidence intervals. Overlaid are univariate GAM-fits with colors representing the type of the curve (yellow = unimodal, blue = increasing). Rao* represents 1/(1-Rao), with Rao being Rao's quadratic entropy[1].

Supplementary Table 1: Criteria used for classification of the relationship between biodiversity and productivity. Curves fitted with GAMs of restricted flexibility (k=3, see methods).

<table>
<tr><th>Curve type</th><th>Significance</th><th>Extrema</th><th>Magnitude</th></tr>
<tr><td>ns</td><td>$R^2 < 0.15$</td><td>-</td><td>-</td></tr>
<tr><td>Concave-</td><td rowspan="4">$R^2 \geq 0.15$</td><td>Minimum at edges, maximum not</td><td>-</td></tr>
<tr><td>Concave+</td><td>Maximum at edges, minimum not</td><td>$\mathrm{Min}(|\mathrm{Biodiv}_{edges} - \mathrm{min}(\mathrm{Biodiv})|) \geq 0.25 \times$ prediction range</td></tr>
<tr><td>Increasing</td><td rowspan="2">Maximum at edges</td><td>$\mathrm{Min}(|\mathrm{Biodiv}_{edges} - \mathrm{min}(\mathrm{Biodiv})|) < 0.25 \times$ prediction range and $\mathrm{Biodiv}_{min(NDVI)} < \mathrm{Biodiv}_{max(NDVI)}$</td></tr>
<tr><td>Decreasing</td><td>$\mathrm{Min}(|\mathrm{Biodiv}_{edges} - \mathrm{min}(\mathrm{Biodiv})|) < 0.25 \times$ prediction range and $\mathrm{Biodiv}_{min(NDVI)} > \mathrm{Biodiv}_{max(NDVI)}$</td></tr>
</table>

R^2 is the explained variance when fitted biodiversity estimates are back-transformed to the original scale.

Supplementary Table 2: Translation of Braun-Blanquet coverage classes[2] into coverage estimates used as species dominance information.

Coverage class	Coverage range (%)	Coverage estimate (%)
1	<1	0.5
2	1-5	3
3	5-25	15
4	25-50	37.5
5	50-75	62.5
6	>75	87.5

Supplementary Table 3: Numbers of community observations and species for the different combinations between ecosystem type and traits considered.

	All ecosystem types	Forests	Grasslands
Communities			
Total	43’229	10’097	11’450
Filtered & all traits	11’172	3324	3738
Filtered & SLA	16’904	4679	5348
Filtered & HGT	26’092	5973	8160
Filtered & SM	14’671	4017	4833
Species			
Total	3402	1642	2000
All traits	1219	809	911
All traits & phylogeny	1210	803	903
SLA	1478	977	1095
SLA & phylogeny	1465	969	1083
HGT	2618	1417	1691
HGT & phylogeny	2589	1401	1671

Supplementary Table 4: Constraints used for classification of relevees as forests or grasslands. Names and numbers correspond to the classification used in Flora Indicativa[3].

Ecosystem type	Original community name	Translated community name	Community #	Constraint
Forests	Waldgesellschaften	forest communities	14	>50%
	Pioniergesellschaften mit Therophyten und Sukkulenten	Pioneer communities with therophytes and succulents	4	
	Alpine Schneetälchengesellschaften	Alpine snowbed communities	8	
	Trockene Magerrasen und Garrigue-Rasen	Dry, nutrient poor meadows and Garrigue-vegetation	9	>50%
	(Kollin-montane) subalpin-alpine Rasen dominiert durch Hemikryptophyten	(colline-montane) subalpine-alpine meadows dominated by hemikryptophytes	10	
	Molinio-Arrhenatheretea		11.2	
	Trifolio-Geranietea		11.1	
Grasslands	*Filipendulo-Convolvuletea*		11.3	<40%
	Epilobietea angustifolii		11.4	
	Mulgedio-Aconitetea		11.5	
	Stickstoffliebende therophytische Pioniergesellschaften	Nitrophilous pioneer communities of therophytes	2	
	Felsspalten- und Schuttgesellschaften	Crevice and scree communities	3	<20%
	Stickstoffliebende Ruderalgesellschaften	Nitrophilous ruderal communities	5	
	Gesellschaften der Flachmoore und Quellen	Communities of bogs and springs	7	

Supplementary References

1. Rao, C. R. Diversity: Its Measurement, Decomposition, Apportionment and Analysis. *Sankhyā Indian J. Stat. Ser. A* **44,** 1–22 (1982).
2. Braun-Blanquet, J. Über den Deckungswert der Arten in den Pflanzengesellschaften der Ordnung Vaccinio-Piceetalia. *Jahresbericht der Naturforschenden Gesellschaft Graubündens* **130,** 115–119 (1946).
3. Landolt, E. *et al. Flora indicativa*. (Haupt Verlag, 2010).
