# Supplementary material for: The productivity-biodiversity relationship varies across diversity dimensions
Source: Nat Commun. 2019 Dec 12;10:5691. doi: 10.1038/s41467-019-13678-1 (PMC6908676; doi:10.1038/s41467-019-13678-1)
Supplement: Supplementary file 3 — Reporting Summary [file 41467_2019_13678_MOESM3_ESM.pdf]

nature research

Corresponding author(s): Philipp Brun

Last updated by author(s): Nov 14, 2019

Reporting Summary

Nature Research wishes to improve the reproducibility of the work that we publish. This form provides structure for consistency and transparency in reporting. For further information on Nature Research policies, see Authors & Referees and the Editorial Policy Checklist.

Statistics

For all statistical analyses, confirm that the following items are present in the figure legend, table legend, main text, or Methods section.

n/a	Confirmed	
☐	☒	The exact sample size (*n*) for each experimental group/condition, given as a discrete number and unit of measurement
☐	☒	A statement on whether measurements were taken from distinct samples or whether the same sample was measured repeatedly
☐	☒	The statistical test(s) used AND whether they are one- or two-sided<br>*Only common tests should be described solely by name; describe more complex techniques in the Methods section.*
☐	☒	A description of all covariates tested
☐	☒	A description of any assumptions or corrections, such as tests of normality and adjustment for multiple comparisons
☐	☒	A full description of the statistical parameters including central tendency (e.g. means) or other basic estimates (e.g. regression coefficient) AND variation (e.g. standard deviation) or associated estimates of uncertainty (e.g. confidence intervals)
☐	☐	For null hypothesis testing, the test statistic (e.g. *F*, *t*, *r*) with confidence intervals, effect sizes, degrees of freedom and *P* value noted<br>*Give P values as exact values whenever suitable.*
☒	☐	For Bayesian analysis, information on the choice of priors and Markov chain Monte Carlo settings
☒	☐	For hierarchical and complex designs, identification of the appropriate level for tests and full reporting of outcomes
☐	☒	Estimates of effect sizes (e.g. Cohen's *d*, Pearson's *r*), indicating how they were calculated

Our web collection on statistics for biologists contains articles on many of the points above.

Software and code

Policy information about availability of computer code

Data collection	Data was obtained from different sources described in the "Data" section of the Methods. Filtering and transformations were done in the R environment.
Data analysis	All analyses were conducted in the R environment. R code generated for the analyses is available from the corresponding author upon reasonable request.

For manuscripts utilizing custom algorithms or software that are central to the research but not yet described in published literature, software must be made available to editors/reviewers. We strongly encourage code deposition in a community repository (e.g. GitHub). See the Nature Research guidelines for submitting code & software for further information.

Data

Policy information about availability of data

All manuscripts must include a data availability statement. This statement should provide the following information, where applicable:

- Accession codes, unique identifiers, or web links for publicly available datasets
- A list of figures that have associated raw data
- A description of any restrictions on data availability

NDVI data from the Landsat mission are available form https://landsat.gsfc.nasa.gov/. Climate data from CHELSA are available from http://chelsa-climate.org/. Plant community data, phylogenetic data, and trait data are available from the corresponding author on reasonable request. The source data underlying the main results (Figs 2a, 3c-e, 4, and 6) are provided as a Source Data file.

Field-specific reporting

Please select the one below that is the best fit for your research. If you are not sure, read the appropriate sections before making your selection.

☐ Life sciences ☐ Behavioural & social sciences ☒ Ecological, evolutionary & environmental sciences

For a reference copy of the document with all sections, see nature.com/documents/nr-reporting-summary-flat.pdf

Ecological, evolutionary & environmental sciences study design

All studies must disclose on these points even when the disclosure is negative.

Study description	We used generalized additive modeling to infer relationship types between biodiversity facets and productivity and we compared biodiversity at high-productivity sites with biodiversity of random assemblages based on overlaps in their frequency distributions.
Research sample	Our raw data consisted of about 43000 plant community observations in the French alps.
Sampling strategy	Within 18 productivity bins we resampled 100 (or 2000 for random assemblages) times 40 community observations under the constraint that they had to be at least 5 km apart from each other.
Data collection	No data was collected in the context of this study.
Timing and spatial scale	The study area incuded the French alps (Figure 2).
Data exclusions	Community observations were excluded when they were incomplete, had a <10 species, unrealistic coverage information or inaccurate spatial information (See subsection 'Community data' in the Methods).
Reproducibility	No experiments were conducted for this study and the Methods include careful descriptions of data considered and analyses conducted.
Randomization	We generated random communities by extending Gotelli's swap algorithm to abundance data (see section 'Constructing null communities' in the Methods).
Blinding	This study did not include an a priori experimental design and thus blinding strategies were not relevant.

Did the study involve field work? ☐ Yes ☒ No

Reporting for specific materials, systems and methods

We require information from authors about some types of materials, experimental systems and methods used in many studies. Here, indicate whether each material, system or method listed is relevant to your study. If you are not sure if a list item applies to your research, read the appropriate section before selecting a response.

Materials & experimental systems

n/a	Involved in the study
☒	☐ Antibodies
☒	☐ Eukaryotic cell lines
☒	☐ Palaeontology
☒	☐ Animals and other organisms
☒	☐ Human research participants
☒	☐ Clinical data

Methods

n/a	Involved in the study
☒	☐ ChIP-seq
☒	☐ Flow cytometry
☒	☐ MRI-based neuroimaging
